# Desmoplastic infantile ganglioglioma

**DOI:** 10.11604/pamj.2019.32.113.12669

**Published:** 2019-03-11

**Authors:** Salma Kamoun, Heifa Azouz, Marwa Zemmali, Slim Haouet, Nidhameddine Kchir

**Affiliations:** 1 Department of Pathology, Rabta Hospital, Faculty of Medicine of Tunis, University of Tunis el Manar, Tunisia; 2 Department of Neurosurgery, National Institute of Neurology, Tunis, Tunisia

**Keywords:** Tumor, central nervous system, infantile desmoplastic ganglioglioma

## Abstract

The term desmoplastic infantile ganglioglioma was coined by VandenBerg et al in 1987. In their first report these authors referred to a rare, distinct brain tumor. About 60 cases of desmoplastic infantile ganglioglioma have been described in the literature since its first description. We report a case of a 6-year-old girl who was admitted for seizure without family history. Magnetic resonance imaging scan showed a hypodense area in the right temporal region. A right temporal craniotomy was performed and the tumor was excised. The pathologic examination revealed the diagnosis of desmoplastic infantile ganglioglioma.

## Introduction

The term desmoplastic infantile ganglioglioma (DIG) was coined by VandenBerg et al in 1987 [1]. In their first report these authors referred to a rare, distinct brain tumor. About 60 cases of DIG have been described in the literature since its first description [2]. In this work, we describe the histologic, immunohistochemical and differential diagnoses of DIG.

## Patient and observation

**Figure 1:**
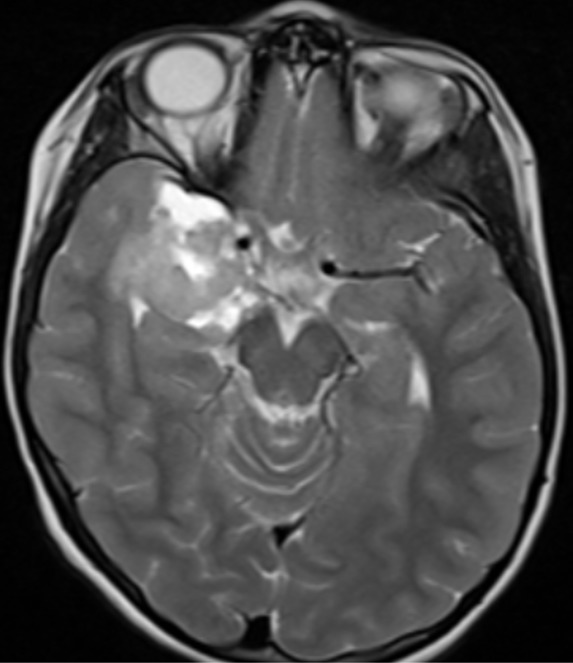
Magnetic resonance imaging scan showing an area with contrast enhancing in the right temporal region

**Figure 2:**
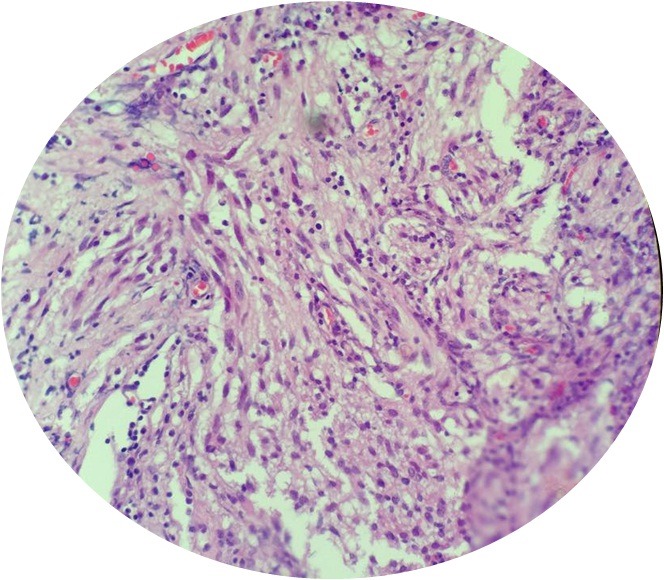
Spindled portion of the tumor, the cells have obviously eosinophilic cytoplasm and glial appearance

**Figure 3:**
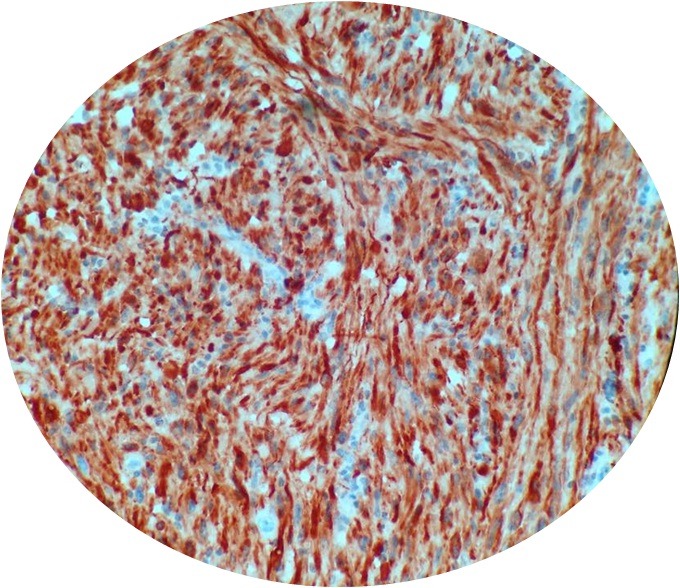
GFAP is strongly positive in the glial portion of the tumor, but negative in blood vessel and mesenchymal cells in the desmoplastic areas

**Figure 4:**
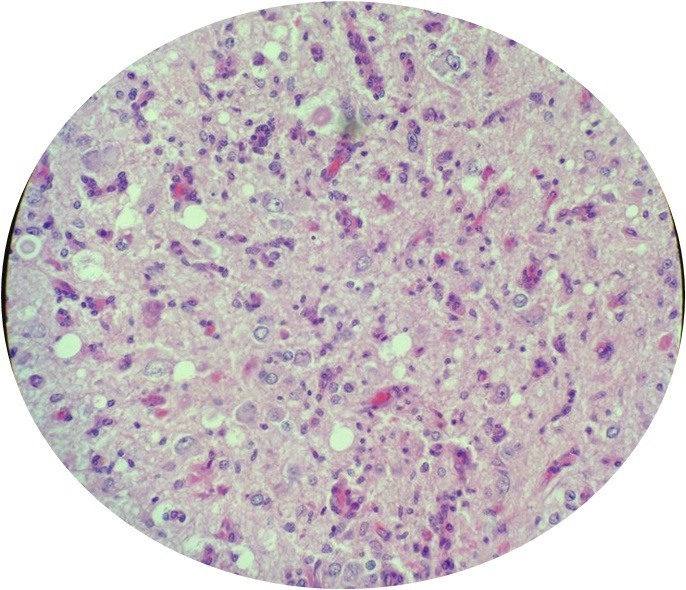
Neuronal portion of tumor, cells range from atypical ganglionic cells to small polygonal cell types

**Figure 5:**
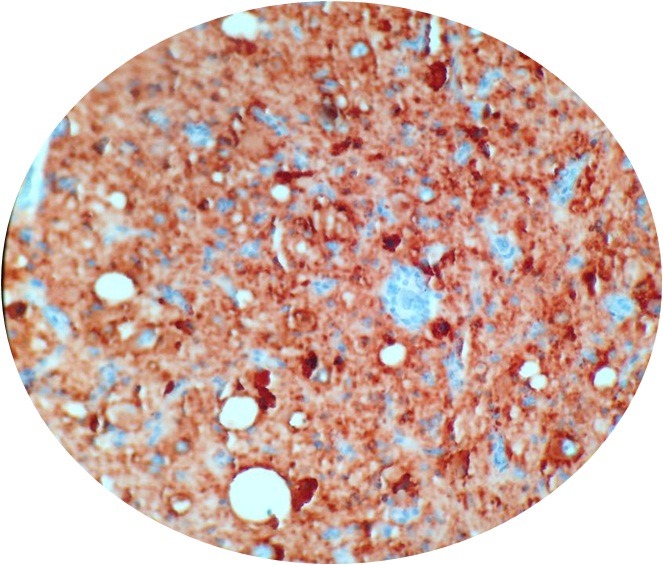
Synaptophysin is positive in ganglion cells

A 6-year-old girl was admitted for seizure without family history. Neurological examination was normal. Magnetic resonance imaging scan showed a hypodense area in the right temporal region, with contrast enhancing solid as well as large cystic components (Figure 1). A right temporal craniotomy was performed and the tumor was excised. The pathologic examination revealed a markedly desmoplastic tumor, showing deposition of dense collagen fibers. The neoplasic cell population was heterogenous, composed of spindle-shaped astrocytes with a fascicular arrangement (Figure 2). These cells show intense immunoreactivity for GFAP (Figure 3). Scattered ganglion cells with copious cytoplasm and irregular nuclei were also observed, indicating neuronal differentiation (Figure 4). These neuronal cells express synaptophysin (Figure 5). No mitosis or necrosis was present.

## Discussion

DIG is a supratentorial brain tumor occurring before 2 years. It has similar clinical and neuroimaging features with desmoplastic infantile astrocytoma (DIA), including a favourable prognosis, but DIA lacks ganglion cells. DIG and DIA have been categorized together as desmoplastic infantile astrocytoma/ganglioglioma in the last editions of the WHO classification. They are WHO grade 1 tumors [3]. The main histologic differential diagnoses are pleomorphic xanthoastrocytoma, gliofibroma, ganglioglioma [4] and other small blue cell tumors of the central nervous system (medulloblastoma, central nervous system primitive neuroectodermal tumors, pineoblastomas) [5]. Total resection is the best treatment and offers long term survival [6]. Dissemination of these tumors through the cerebrospinal fluid has been reported, but is rare event [7]. Anaplastic histological features as high mitotic rate, microvascular proliferation and perinecrotic palisading tumor cells had no influence over survival [3, 8].

## Conclusion

DIA and DIG are tumors of the same family. They occur in children under 2-year-old. Their diagnosis should be rendered only after correlation between neuropathologic, clinical and neuroimaging features. They have favorable prognosis if completely resected.

## Competing interests

The authors declare no competing interest.
